# Anticancer Efficacy and Mechanisms of a Dual Targeting of Norepinephrine Transporter and Thyrointegrin αvβ3 Antagonist in Neuroblastoma

**DOI:** 10.7150/jca.72522

**Published:** 2022-05-16

**Authors:** Kavitha Godugu, Ozlem O. Karakus, Kazutoshi Fujioka, Gennadi V. Glinsky, Shaker A. Mousa

**Affiliations:** 1 The Pharmaceutical Research Institute, Albany College of Pharmacy and Health Sciences, Rensselaer, New York 12144, United States.; 2 Institute of Engineering in Medicine, University of California San Diego, San Diego, CA.

## Abstract

**Background:** In neuroendocrine tumors, the norepinephrine transporter (NET) is very active and has been exploited for diagnostic imaging purposes and/or therapy with localized radiotherapy. Integrin αvβ3 is generously expressed by and/or activated on cancer cells, but not by nonmalignant cells.

**Purpose:** In the present investigation, the anticancer efficacy of the dual targeting of norepinephrine transporter (NET), benzylguanidine (BG), and thyrointegrin αvβ3 receptors antagonist triazole tetraiodothyroacetic acid (TAT) conjugated via the non-cleavable linker polyethylene glycol (P, PEG400) in the treatment of human neuroblastoma was evaluated.

**Experimental approach:** The synthesized dual targeting compound, a novel new chemical entity named BG-P_400_-TAT, has purity > 98% and was formulated and tested in neuroblastoma models using neuroblastoma cell lines (SK-N-FI, SMS-KCN and SMS-KANR) implanted in SCID and NSG mice models.

**Key Results:** BG-P_400_-TAT demonstrated significant (**P<0.01, ***P< 0.001) suppression of neuroblastoma tumor progression, growth, and viability in both mice models implanted with the neuroblastoma. The pharmacokinetic and biodistribution profile of BG-P_400_-TAT showed a significant increase in BG-P_400_-TAT levels in plasma and xenografts of NSG compared to SCID mice. Further our RNAseq genome-wide expression profiling experiments in neuroblastoma cell line SKNAS results showed that BG-P_400_-TAT treatment altered the signal transduction pathways, intracellular multiprotein complexes and Independent GSEA.

**Conclusion & Implications:** BG-P_400_-TAT represents a potential lead candidate for the treatment of neuroblastoma and other neuroendocrine tumors.

## Introduction

Neuroblastoma (NB) is the second most common solid malignancy in children, and it arises from neural crest cells. It accounts for approximately 8% of all childhood cancers and 15 % of childhood cancer mortality 1. Unique features of neuroblastoma tumors are dependent on widely varying factors, including age, disease stage, tumor site, biological features, and the high frequency of metastatic disease at diagnosis and the tendency for spontaneous regression of tumors in infancy. Even with intensive therapy, the prognosis for neuroblastoma patients has not significantly improved in contrast to other pediatric tumors 2. Hence, there is a desperate need for novel therapies to improve survival and to decrease morbidity.

Neuroblastoma is unique among pediatric cancers in expression of norepinephrine transporter (NET). Previous studies showed that NET can be targeted for imaging as well as for therapy 3. Meta-iodobenzylguanidine (MIBG) and the guanethidine analog of norepinephrine are widely used theranostic agents targeting NET in neuroblastoma 4. However, the studies showed that MIBG radiotherapy remains suboptimal, and the outcome leads to relapse from undetectable tumors.

Integrins are proteins on the extracellular domain of the plasma membrane. Among the 24 integrin isoforms, integrin αvβ3 is highly expressed on tumor cells and dividing blood vessels 5, and it has a receptor for thyroid hormone. It is well documented that the interaction between thyroid hormones (L-thyroxine (T_4_) and 3,5,3'-triiodo-L-thyronine (T_3_) promotes cell proliferation, tumor angiogenesis, and organ-specific metastasis in various types of cancers including breast, lung, glioma, pancreatic, and neuroblastoma, respectively 6-10.

Our previous studies reported that a deaminated analog of thyroid hormone, tetraiodothyroacetic acid (tetrac), can block signal transduction mechanisms and mediate the promotion of cancer cell proliferation 11, 12. A dual targeting novel ligand, BG-P_400_-TAT and derivatives, where P is polyethylene glycol and TAT is triazole tetraiodothyroacetic acid , can bind to integrin αvβ3, and benzylguanidine (BG) targets NET receptors on neuroblastoma cells 13, 14.

The use of Immunodeficient mice for developing xenograft models of human cancer has extensively reviewed. To date for developing neuroblastoma xenografts extensively used mouse strain are SCID mice, SCID, beige mice and nude mice. All these mouse strains are T-cell deficient and exhibit residual immune formation with natural killer cells that can inhibit tumor formation and metastatic spread. Recently NSG (NOD/SCID interleukin-2 receptor null) mice have shown to be one of the most immunodeficient mouse, deficient in both T, B lymphocytes and NK cells and display impaired dendritic cell functions. Several studies reported the use of NSG mice as NB xenograft model was advantageous in therapeutic and survival studies compared to SCID and nude mice. However, it is important to study the intrinsic tumorigenic properties of NB cells injected into NSG mice, in order to validate the relevant xenograft model to study human-specific tumor therapies.

In the current study, we examined and compared tumorigenic effects of BG-P_400_-TAT in neuroblastoma cell line SK-N-FI, SMS-KCN and SMS-KANR in SCID versus NSG mice. Further, we evaluated the anticancer effect of BG-P_400_-TAT of neuroblastoma xenografts in both mice models. In addition, we evaluated the pharmacokinetics and molecular mechanisms of BG-P_400_-TAT.

## Materials and Methods

### Materials

Benzylguanidine-PEG-TAT (BG-P_400_-TAT) was synthesized at the Pharmaceutical Research Institute 13. Iscove's Modified Dulbecco's medium (IMDM), fetal bovine serum (FBS), penicillin, streptomycin, and trypsin/EDTA were purchased from Sigma-Aldrich (St. Louis, MO, USA). The neuroblastoma cell line SK-N-FI, SMS-KCN and SMS-KANR were obtained from the COG-NB cell repository and were authenticated by COG (http://www.cogcell.org/clid.php). Acetonitrile, methanol, water, and formic acid were LC-MS grade and purchased from Sigma-Aldrich.

### Cell culture

All the cells were grown in IMDM supplemented with 10% FBS, 1% penicillin, and 1% streptomycin. Cells were cultured at 37 °C to sub-confluence and treated with 0.25% (w/v) trypsin/EDTA to induce cell release from culture flasks. Cells for grafting were washed with culture medium, suspended in IMDM that was free of phenol red and FBS and subjected to counting.

### Animals and xenografts

Immunodeficient female SCID mice and NOD-Prkdc^scid^ IL2rg^null^ (NSG) mice aged 5-6 weeks and weighing 18-20 g were purchased from Taconic Laboratories (Germantown, NY, USA). All animal studies were conducted at the animal facility of the Veteran Affairs (VA) Medical Center, Albany, NY, USA in accordance with current institutional guidelines for humane animal treatment and approved by the VA IACUC. Mice were maintained under specific pathogen free conditions and housed under controlled conditions of temperature (20-24 °C) and humidity (60-70%) and 12 h light/dark cycle. Animals were fed a standard pelleted mouse chow and allowed to acclimatize for 5 days before the study.

### Neuroblastoma cancer xenografts

For the subcutaneous neuroblastoma cancer tumor model, SK-N-FI, SMS-KCN and SMS-KANR neuroblastoma cells were harvested, suspended in 100 μL of IMDM with 50% Matrigel^®^, and 1 × 10^6^ cells were implanted subcutaneously dorsally in each flank to achieve two independent tumors per animal (n=40). Immediately prior to initiation of treatments, animals were randomized into treatment groups (5 animals/group) by tumor volume measured with Vernier calipers. Treatments were begun after detection of a palpable tumor mass (4-5 days post implantation). The treatments were control (PBS) and BG-P_400_-TAT (10 mg/kg body weight). The treatments were administered daily, subcutaneously on the ventral side of the animal, for 21 days. Then the animals were terminated, and tumors were collected and fixed in 10% formalin.

### Histopathology

The fixed samples were placed in cassettes and dehydrated using an automated tissue processor. The processed tissues were embedded in paraffin wax and the blocks trimmed and sectioned to about 5 × 5 × 4 µm size using a microtome. The tissue sections were mounted on glass slides using a hot plate and subsequently treated in the order of 100%, 90% and 70% ethanol for 2 min each. Finally, the tissue sections were rinsed with water, stained with Harris's hematoxylin and eosin (H &E), and examined under a light microscope.

### Pharmacokinetics

For the pharmacokinetics study, SCID (n=5) and NSG mice (n=5) were administrated subcutaneously with BG-P_400_-TAT (10 mg/kg) and plasma was collected at 0.5, 1, 2, 4, 6, 12, and 24 h. Plasma samples were stored at -80 °C for further analysis.

#### Sample preparation of plasma and tumor using liquid-liquid extraction

Fifty μL of plasma with or without BG-P_400_-TAT (150 ng/μL) and internal standard (IS) reserpine (50 ng/μL) were vortex-mixed with 1 mL of acetonitrile/methanol (50/50 v/v) for 30 min. Tumor samples were thawed, weighed, and homogenized with the same volume of ice-cold water with a hand homogenizer. Tumor homogenate (100 μL) with or without BG-P_400_-TAT and IS was vortex-mixed with 1 mL of acetonitrile/water (80/20 v/v) for 30 min. After centrifugation, solvent was evaporated under a nitrogen stream at 50°C. Extracts were reconstituted with 50 μL of acetonitrile/water (80/20 v/v).

### LC-MS/MS analysis

An API-4000 mass spectrometer (Sciex, Framingham, MA, USA) equipped with Shimadzu UPLC system (Kyoto, Japan) was used for LC-MS/MS analyses. A Kinetex 2.6 μm Polar C18 100 LS column (50 × 2.1 mm, Phenomenex, Torrance, CA, USA) was used for reversed-phase separation. Mobile phases were (A) water containing 0.1% formic acid and 5% acetonitrile and (B) acetonitrile with 0.1 % formic acid. The flow rate was 0.5 mL/min, and the gradient was linear from 0% B for 1 min to 95% B for 7-9 min. The oven temperature was 40°C and the injection volume was 5 μL. Electro-spray ionization (ESI) was used in positive MRM mode. Mass transitions for analytes were: Q1/Q3: 1285.0/133 (BG-P_400_-TAT); 609.4/195.0 (IS). A standard curve was obtained using standard solutions of BG-P_400_-TAT with concentrations of 3000, 1000, 300, 100, 30, and 10 ng/mL by the internal standard method.

### Molecular Mechanisms

#### RNA isolation from neuroblastoma cells and processing for microarrays

Cells were cultured in 50 cm² cell culture flasks with 10 ml phenol red free IMDM supplemented with 10% FBS, 1% penicillin, and 1% streptomycin. During the passaging, cells were cultured at 37 °C to sub-confluence and treated with 0.25% (w/v) trypsin/EDTA to induce cell release from culture flasks. Cells were treated (at 50% confluence) with 30 µM of the BG-P_400_-TAT for 48 hours. Total RNA was immediately isolated from harvested cells using Trizole and checked for quality using a Bioanalyzer before being utilized for microarray analysis. The quality and the concentrations of the extracted RNA was analyzed using the Nanodrop (Thermo Fisher Scientific, Waltham, MA) and Agilent Bioanalyzer (Agilent Technologies, Santa Clara, CA). RNA samples (100 ng) deemed to be of sufficient quality (RIN greater than 8) were processed according to the standard Affymetrix RNA labeling protocol. Three independent biological replicates of control and treated samples were concurrently interrogated in gene expression profiling analyses. In preliminary experiments, the treatment dose and duration were carefully selected not to significantly affect growth and survival of target cells during the duration of experiments.

#### Gene expression profiling analysis, data retrieval and analytical protocols for identification of differentially expressed genes

Gene expression profiling experiments were performed using both microarray and RNAseq analytical platforms. Samples were processed according to the standard Affymetrix RNA labeling protocol. Labeled RNA samples were processed for hybridization employing the Clariom™ S human array platform (Affymetrix, Santa Clara, CA) at the Center for Functional Genomics, University at Albany, Rensselaer, NY. Briefly, 100 ng of total RNA was processed using the WT Plus Reagent kit (Affymetrix). Sense target cDNAs were generated using the standard Affymetrix WT protocol and hybridized to Affymetrix Human Clariom S arrays. Arrays were washed, stained, and scanned on a GeneChip 3000 7G scanner using Affymetrix GeneChip Command Console Software (AGCC). Transcriptome Analysis Console Software (TAC v3.0.1.5) was used to identify differentially expressed genes (DEGs). Briefly, the CEL files were summarized using the SST-RMA algorithm in TAC and the normalized data were subjected to one-way ANOVA with a Benjamin Hochberg False Discovery Rate correction included (p<0.05). A 1.5-fold expression change cut-off was used to select entities that were statistically differentially expressed between the conditions being compared (treated and untreated groups). In the standard workflow protocol, the fragmented biotin-labeled cDNAs were hybridized for 16 h to Affymetrix Arrays, scanned on an Affymetrix Scanner 3000 7G using AGCC software, and processed as described above. Alternatively, CEL files after QC screening using Affymetrix Expression Console software were imported into GeneSpring GX11.5 (Agilent Technologies). The data was then quantile normalized using PLIER and baseline transformed to the median of the control samples. The probe sets were further filtered to exclude the bottom 20th percentile across all samples. The resulting entity lists were subjected to an unpaired T-test with the Benjamini-Hochberg False Discovery Rate (FDR) correction and a 1.5-fold expression changes filter to identify differentially expressed transcripts between the control and test conditions at a p-value <0.05. During the selection of differentially expressed genes, both nominal and FDR adjusted p-values were considered. All analyzed and reported data are MIAME compliant and the raw data have been deposited in Gene Expression Omnibus (GEO) as detailed on the Microarray Gene Expression Data Society (MGED) society website (http://www.mged.org/Workgroups/MIAME/miame.html). Overall, the workflow of the microarray analyses was modeled based on previously published contributions 15-17.

Gene set enrichment analyses of differentially expressed genes (DEGs) were carried-out using the Enrichr bioinformatics platform, which enables the interrogation of nearly 200,000 gene sets from more than 100 gene set libraries. The Enrichr API (January 2018 through April 2021 releases) 18, 19 was used to test genes of interest for significant enrichment in numerous functional categories. When technically and analytically feasible, different sets of DEGs defined at multiple significance levels of statistical metrics and comprising from dozens to several thousand individual genetic loci were analyzed using differential Gene set enrichment analysis (GSEA) to gain insights into biological effects of DEGs and infer potential mechanisms of anticancer activities. This approach was successfully implemented for identification and characterization of human-specific regulatory networks governed by human-specific transcription factor-binding sites 20-24 and functional enhancer element 25-27, 13,824 genes associated with 59,732 human-specific regulatory sequences 28, 8,405 genes associated with 35,074 human-specific neuroregulatory single-nucleotide changes 29, and 8,384 genes regulated by stem cell-associated regulatory sequences^30^. Initial GSEA entail interrogations of each specific set of DEGs using 29 distinct genomic databases, including comprehensive pathway enrichment Gene Ontology (GO) analyses followed by in-depth analyses of the selected genomic databases deemed most statistically informative. In all tables and plots (unless stated otherwise), in addition to the nominal p values and adjusted p values, the “combined score” calculated by Enrichr software is reported, which is a product of the significance estimate and the magnitude of enrichment (combined score c = log(p) * z, where p is the Fisher's exact test p-value and z is the z-score deviation from the expected rank).

### Statistical Analysis

Statistical values reported were calculated using Student's *t* test for pared comparison and for comparison between two or more sets of data, ANOVA was used with GraphPad Prism 7 software (GraphPad, San Diego, CA, USA). *P< 0.05, **P< 0.01, ***P< 0.001 were considered statistically significant values.

## Results

### Tumorigenesis rates between SCID and NSG mice

**Figure 1:**
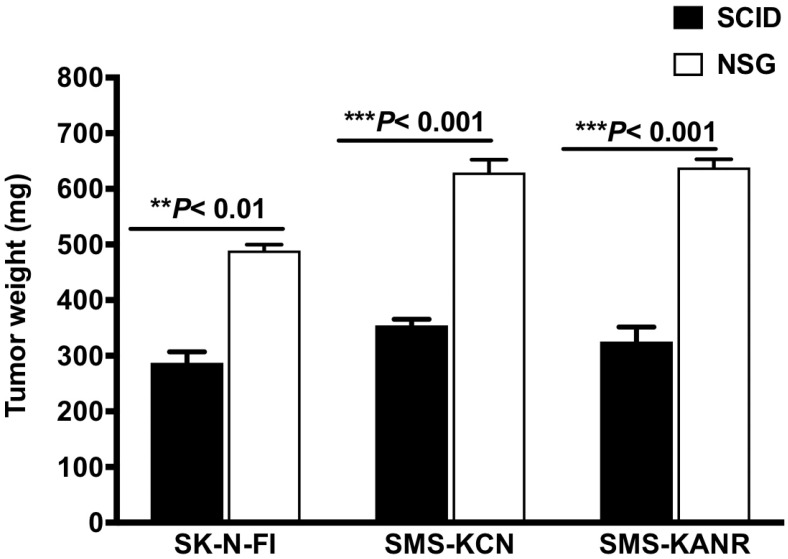
Tumor growth after 21 days in SCID and NSG mice implanted with neuroblastoma cells (SK-N-FI, SMS-KCN and SMS-KANR). Values are presented as mean ± S.D. **P< 0.01, No significance (NS).

SCID and NSG mice were subcutaneously injected with the neuroblastoma cells and tumorigenesis rates were investigated after 21 days. In SCID mice the tumor weight decreased by 59%, 56% and 51% for SK-N-FI, SMS-KCN and SMS-KANR xenografts. The difference was statistically significant (**P< 0.01, ***P< 0.001) compared to NSG mice (Figure 1).

### Effect of BG-P_400_-TAT on neuroblastoma xenografts

**Figure 2:**
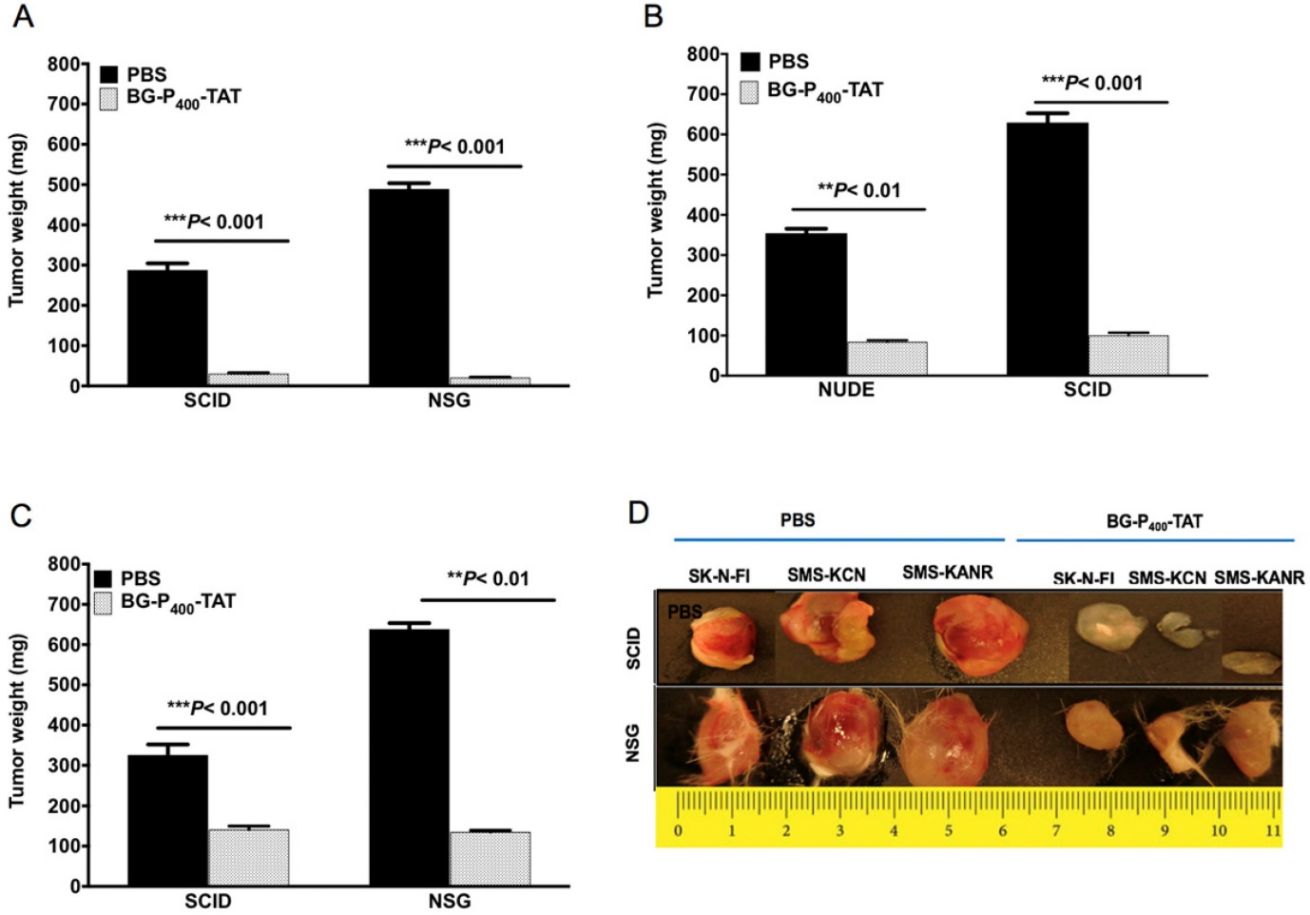
Effect of BG-P_400_-TAT on tumor growth in SCID and NSG mice. Xenografted mice were administrated subcutaneously daily with control (PBS) or BG-P_400_-TAT (10 mg/kg) for 21 days. Tumor weights are shown for **A)** SK-N-FI; **B)** SMS-KCN; **C)** SMS-KANR. **D)** Representative images of tumor size. Values are presented as mean ± S.D. **P< 0.01, ***P< 0.001, compared to respective controls.

We next compared the anticancer effect of xenografts treated with BG-P_400_-TAT (10 mg/kg) in the SCID and NSG mice. BG-P_400_-TAT significantly decreased tumor weight in SK-N-FI, SMS-KCN and SMS-KANR xenografts in the SCID mice by 90%, 76% and 57%, respectively, and in the NSG mice by 96%, 84% and 79% respectively, when compared to their respective controls (Figure 2).

**Figure 3:**
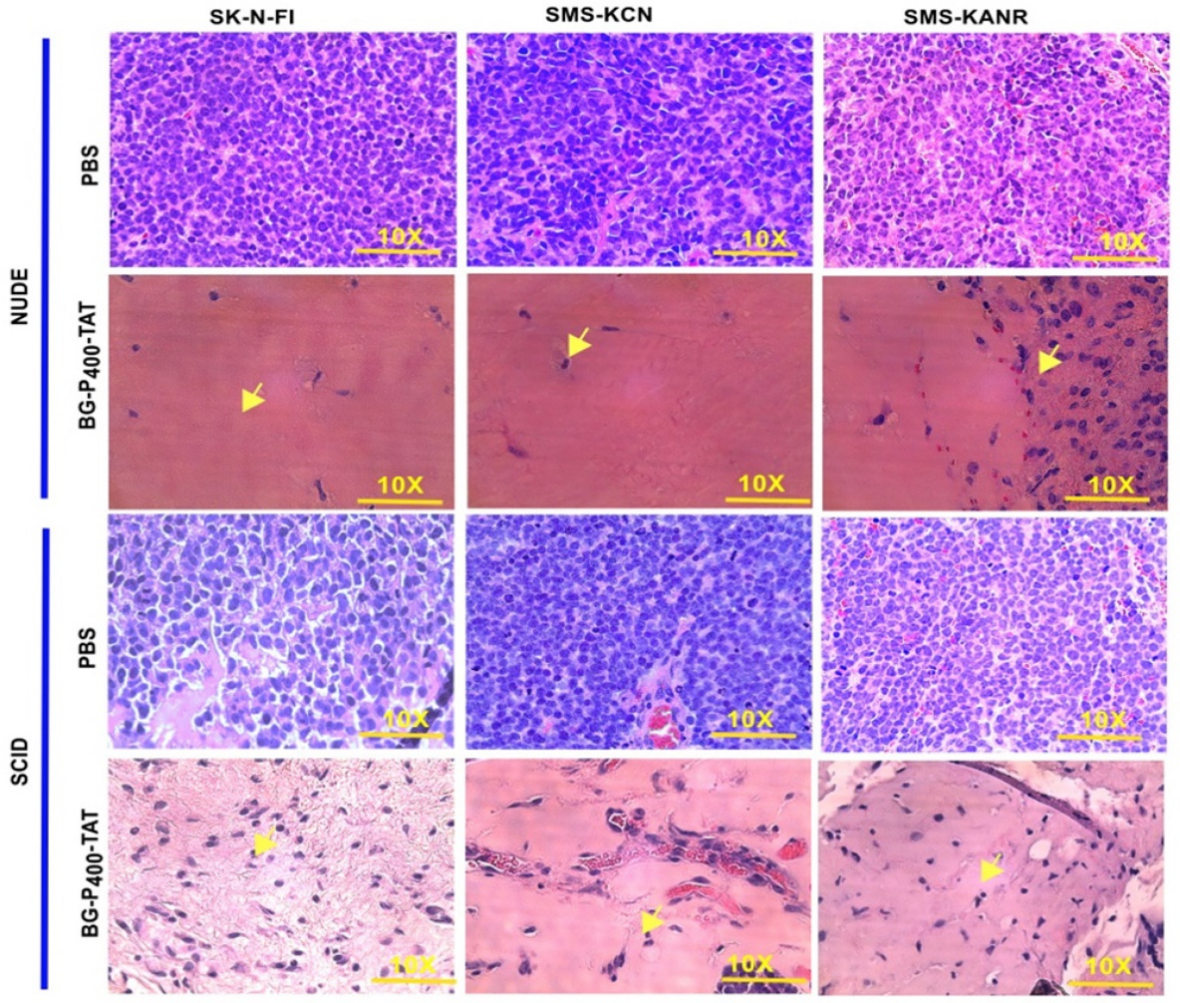
Histopathological images of H&E staining of xenografts after 21 days of treatment with control (PBS) and BG-P_400_-TAT (10 mg/kg) in SCID and NSG mice. The scale bar is 10X. Complete infiltration (~100%) with the malignant cells was observed in controls. In treatment groups, necrosis, and hyalinization >90% were observed. Green arrows indicate fibroblasts, black arrow indicates blood vessels with red blood cells.

To compare the histopathological changes in the neuroblastoma xenografts of the untreated and treated groups, tumors were harvested, fixed, and stained with H&E. In the untreated groups, the staining showed viable tumor cells. On the other hand, in the BG-P_400_-TAT treated groups, there were large areas (> 90%) of hyalinization observed in SCID mice (Figure 3A) and NSG mice (Figure 3B).

### Bioavailability and pharmacokinetics

**Figure 4:**
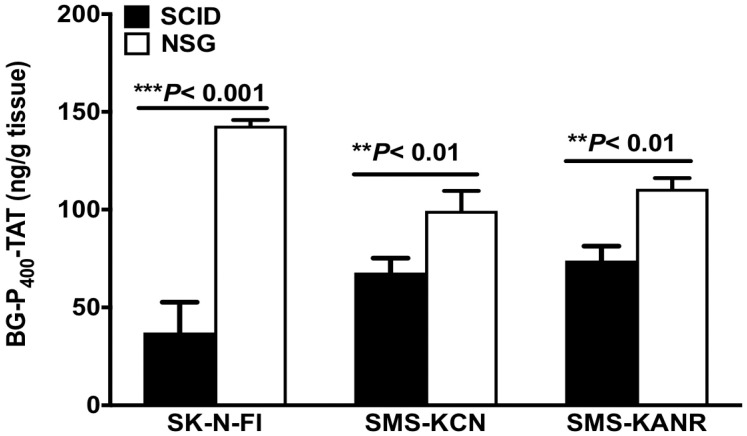
Levels of BG-P_400_-TAT in tumors of SCID and NSG mice. Tumors were collected after 21 days of daily, subcutaneous administration of BG-P_400_-TAT (10 mg/kg). The levels of BG-P_400_-TAT in tumors were quantified using LC-MS/MS. Values are presented as mean ± S.D. **P< 0.01, ***P< 0.001, compared to respective controls, No significance (NS).

Levels of BG-P_400_-TAT in tumors of SCID and NSG mice were determined by using a quantitative bioanalytical LC-MS/MS method. Figure 4 illustrates a significant decrease in the levels of BG-P_400_-TAT in SCID mice implanted with the neuroblastoma cells including SK-N-FI, SMS-KCN and SMS-KANR cells. A significant increase (***P< 0.001) was observed in the levels of BG-P_400_-TAT in NSG mice implanted SK-N-FI, SMS-KCN and SMS-KANR compared to NSG mice.

**Figure 5:**
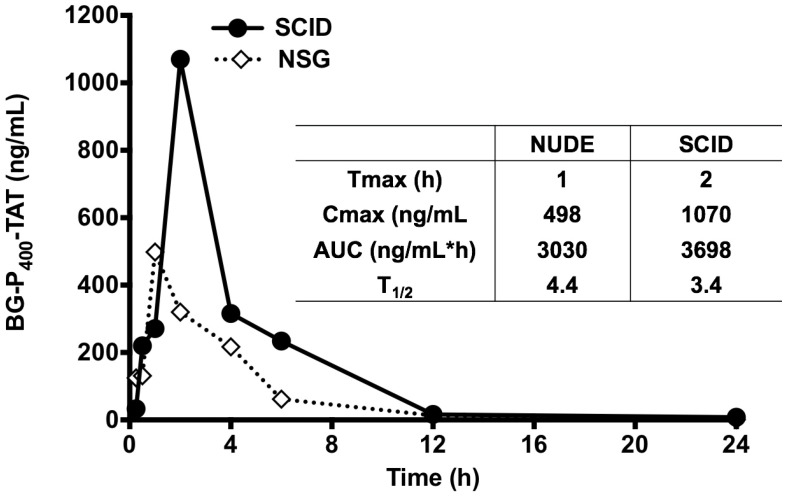
Levels of BG-P_400_-TAT in plasma of SCID and NSG mice. Time course of BG-P_400_-TAT levels in plasma (Left) and PK parameters (Right). Plasma was collected at 0, 2,4 after subcutaneous administration of BG-P_400_-TAT (10 mg/kg). The levels of BG-P_400_-TAT in plasma were quantified using LC-MS/MS. Values are presented as mean ± S.D.

Further, the LC-MS/MS bioanalytical method was applied to PK studies in the mice. The time course of BG-P-TAT levels in mouse plasma is shown in Figure 5. Tmax values ranged from 1 to 2 h in the mice, although the Cmax values in SCID and NSG mice were different, the AUC values in the mice were similar, suggesting similar PK profiles. Half-lives ranged from 3.4 to 4.4 h in the mice, suggesting that BG-P_400_-TAT does not bioaccumulate due to repeated administration.

### Molecular Mechanisms

#### Microarray-miRNA and mRNA expressing profiling

To explore molecular mechanisms of anti-cancer activities of BG-P_400_-TAT, genome-wide expression profiling experiments were carried out (see Methods). Human neuroblastoma SKNAS cells were treated with 30 µM of the BG-P_400_-TAT for 48 hours, harvested, and immediately processed for RNA isolation from three biological replicates of control and drug-treated cells. In a preliminary set of experiments evaluating cell growth and viability, the treatment dose and duration were carefully chosen not to significantly affect growth and survival of target cells during the duration of experiments. Gene expression profiling experiments identified 216 differentially regulated genes (DEGs) in human neuroblastoma cells treated with BG-P_400_-TAT for 48 hrs compared to control vehicle-treated cells. Among 216 DEGs, 150 genes were downregulated, and 66 genes were upregulated.

**Figure 6:**
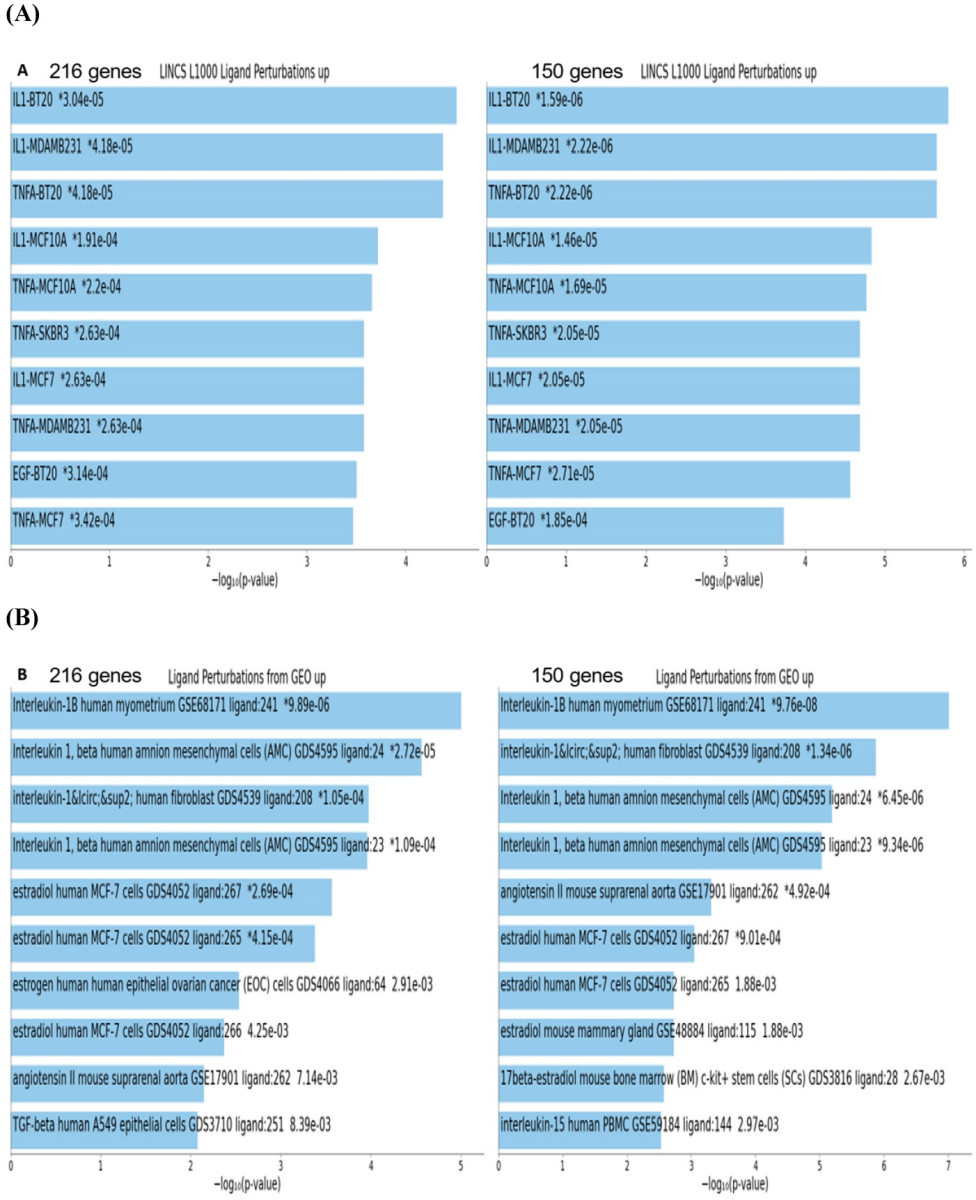
Gene set enrichment analyses of the BG-P_400_-TAT regulated genes identified in human neuroblastoma cells. There are 216 differentially regulated genes identified by the RNAseq genome-wide expression profiling experiments in human neuroblastoma cells SKNAS treated with BG-P_400_-TAT for 48 hrs (150 genes were down-regulated and 66 genes were up-regulated). Independent GSEA were carried out on sets of 216; 150; and 66 genes using 29 genomic databases. Most significantly enriched records identified by the Enrichr bioinformatics platform are reported for 216 genes and 150 genes using different visualization tools (see text for details).

**Figure 7:**
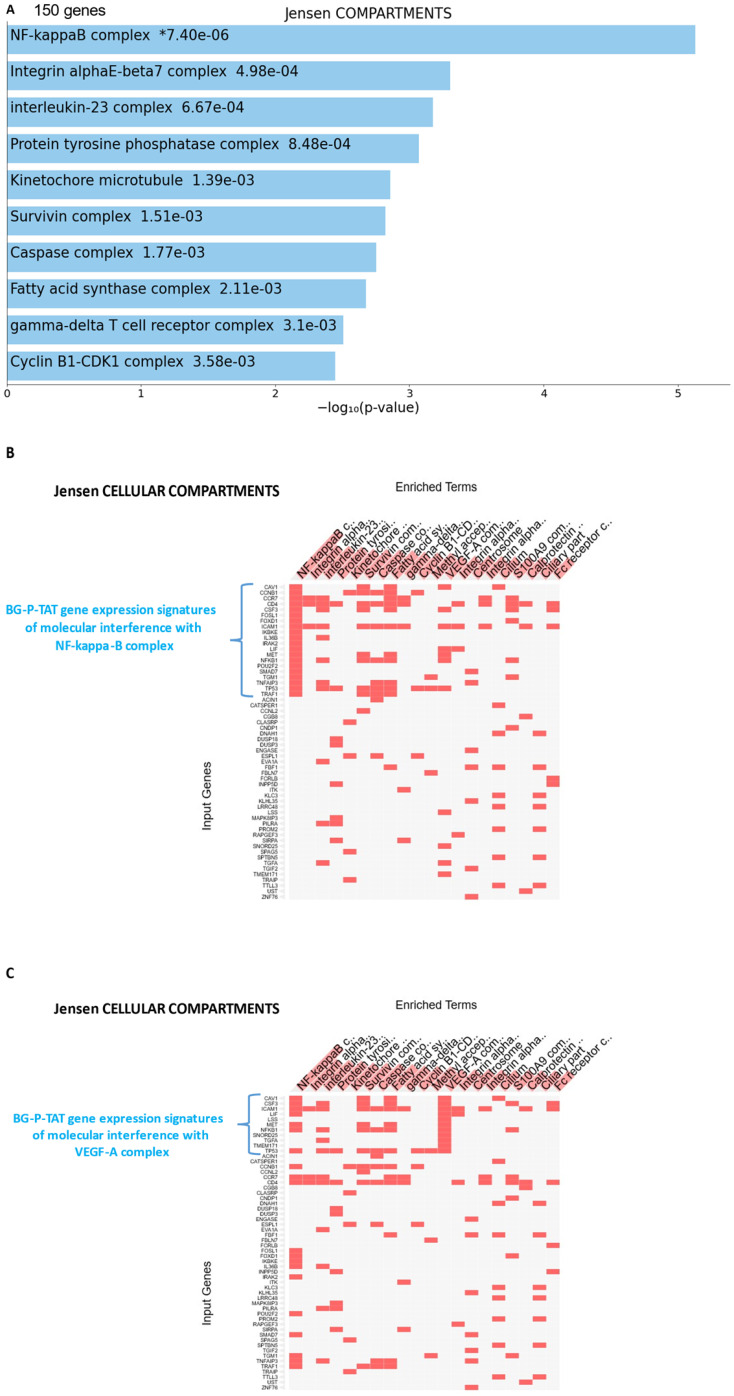

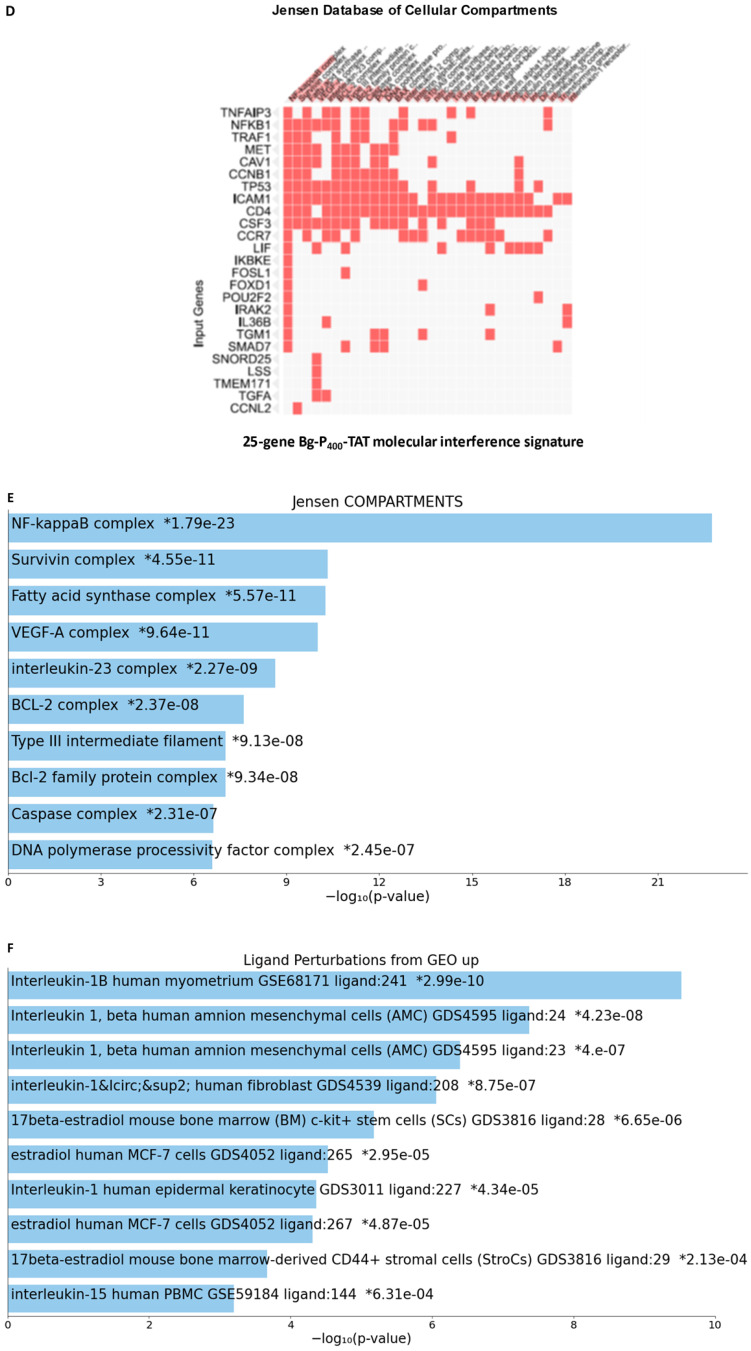
25-gene BG-P_400_-TAT molecular interference signature recapitulates most significantly enriched records of signal transduction pathways and intracellular multiprotein complexes targeted by BG-P_400_-TAT in human neuroblastoma cells. Independent GSEA were carried out on sets of 150 and 25 genes using 29 genomic databases. Most significantly enriched records identified by the Enrichr bioinformatics platform are reported for 150 genes **(A-C)** and 25 genes **(D-F)** using different visualization tools (see text for details).

To gain insights into biological and molecular functions of genes expression of which was significantly affected in neuroblastoma cells by the BG-P_400_-TAT treatment, gene set enrichment analyses (GSEA) of 216 DEGs, 66 up-regulated DEGs, and 150 down-regulated DEGs were carried-out using the Enrichr bioinformatics platform applied to 29 genomics databases (see Methods). We reasoned those systematic comparisons of the levels of statistical significance of enrichment of expression signatures comprising genes affected by BG-P_400_-TAT treatment in human neuroblastoma cells would help to identify gene expression pathways and signaling networks representing candidate molecular targets of BG-P_400_-TAT anti-cancer activities. GSEA identified several significantly enriched records for each category of the BG-P_400_-TAT-regulated genes, among which the LINCS L1000 Ligand Perturbations database (up-regulated genes) and Ligand Perturbations from GEO database (up-regulated genes) had most significantly enriched entries (Figure 6). In contrast, both LINCS L1000 Ligand Perturbations database and Ligand Perturbations from GEO database for down-regulated genes had no significantly enriched records. Because both databases report genes expression of which is up- or down-regulated in response to challenge by endogenous ligands (e.g., growth factors, cytokines, chemokines, etc.), these observations indicate that BG-P_400_-TAT alters the expression of genes that are activated during cellular responses to multiple endogenous ligands. Notably, GSEA identified significant enrichments of endogenous ligands-activated genes among BG-P_400_-TAT down-regulated genes only (Figures 6 & 7), consistent with the hypothesis that BG-P_400_-TAT interferes with signal transduction pathways engaged during cellular responses to endogenous ligands. Signal transduction pathways most prominently targeted by the BG-P_400_-TAT were intracellular signaling pathways activated during cellular responses to IL-1β, TNFα, EGF, and estrogens (Figures 6 & 7). In contrast, GSEA of 66 up-regulated genes identified a single significantly enriched record (Gene Perturbations from GEO database of up-regulated genes) of 8 genes (*COLEC12; MAF; ITGA4; ZNF649; PMCH; RASSF9; CXORF57; SMIM10*) expression of which increased in the *MYC* knockout cells (suggesting that the BG-P_400_-TAT treatment may interfere with MYC pathway by enhancing expression of MYC-down-regulated genes.

GSEA focused on BG-P_400_-TAT down-regulated genes identified genes encoding protein components of the NF-kB complex as the most significantly enriched record reported in the Jensen Cellular Compartments database (Figure 7). Follow-up analyses identified 25-gene BG-P_400_-TAT molecular interference signature that recapitulated most significantly enrichment records captured by GSEA of all BG-P_400_-TAT down-regulated genes (Figure 7) and highlighted multiple additional significantly enriched molecular targets of the BG-P_400_-TAT activity. For example, there are 91 significantly enriched records of intracellular multiprotein complexes reported in the Jensen Cellular Compartments database that appear targeted by the BG-P_400_-TAT treatment (Figure 7).

**Figure 8:**
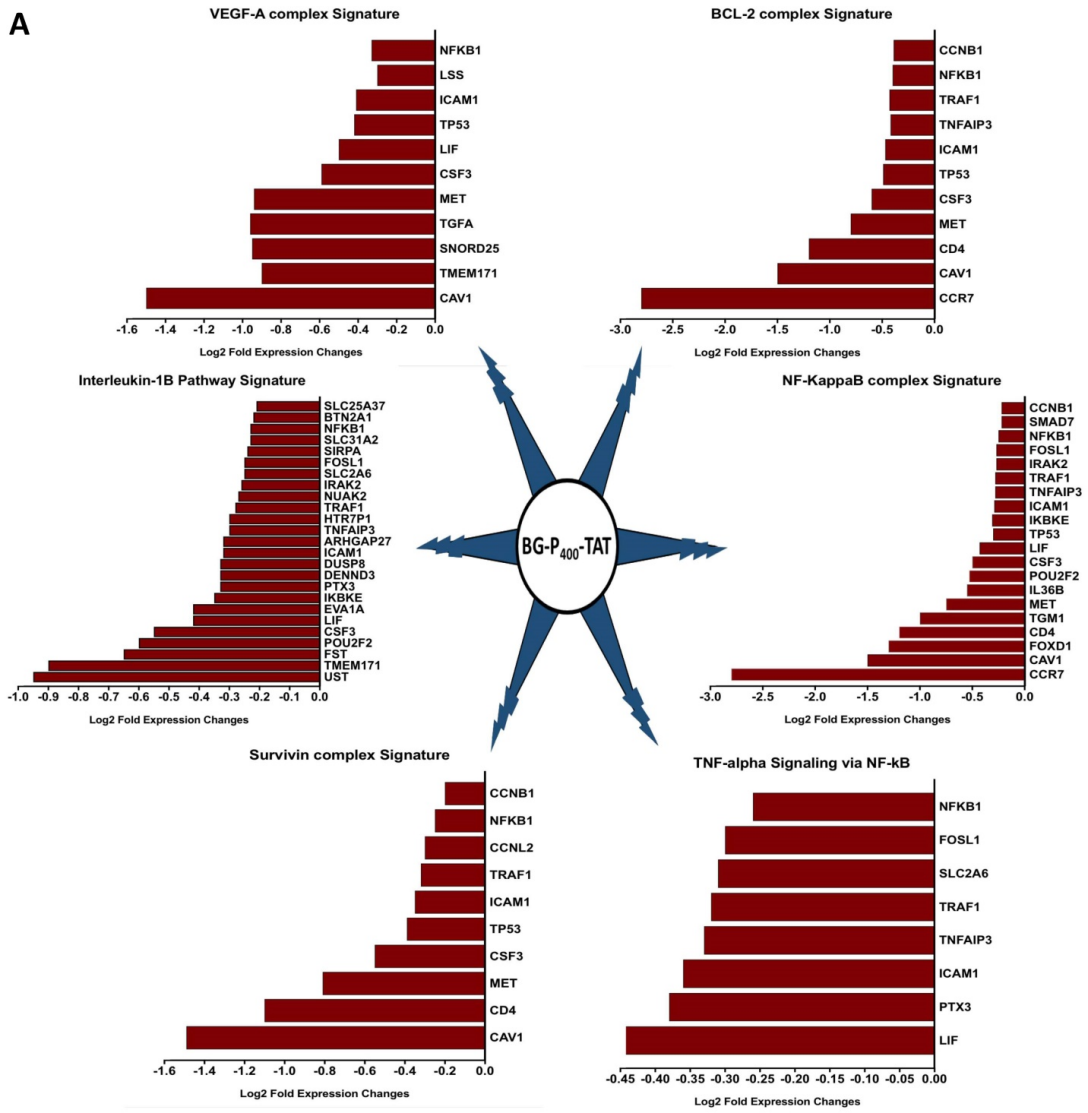

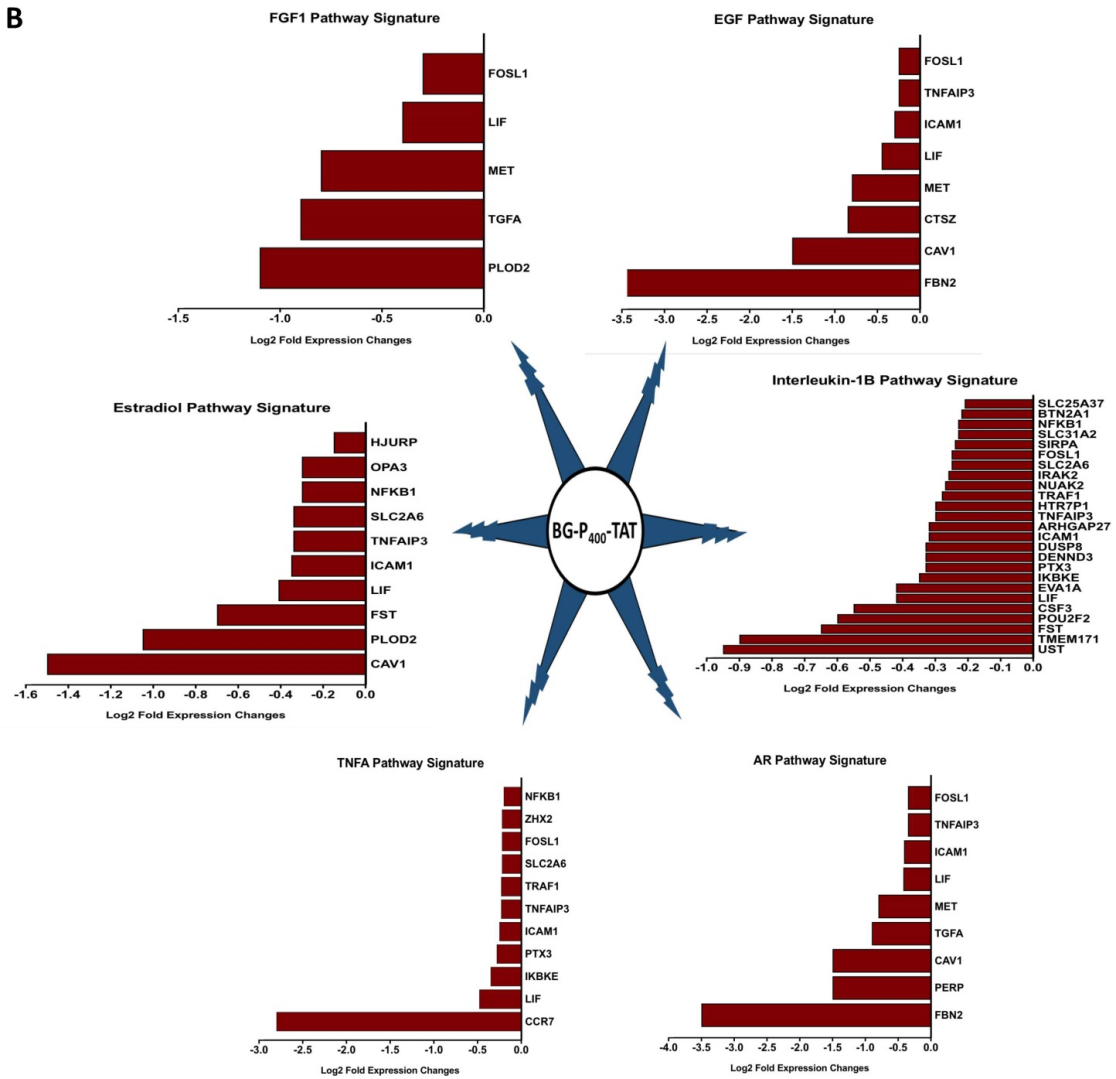
BG-P_400_-TAT induced molecular interference gene expression signatures in human neuroblastoma. Sets of BG-P_400_-TAT down-regulated genes associated with indicated significantly enriched records of intracellular multiprotein complexes **(A)** and signal transduction pathways **(B)** were identified by GSEA, corresponding log2 transformed values of changes in gene expression were retrieved and plotted for visualization.

The graphical summary reporting most significantly enriched records of BG-P_400_-TAT regulated genes in human neuroblastoma cells is shown in the Figure 8. These observations highlight the top-ranked intracellular molecular complexes (Figure 8A) and intracellular signal transduction pathways triggered by exposures to endogenous ligands (Figure 8B) that represent most likely targets of the BG-P_400_-TAT-induced molecular interference associated with its anti-cancer activities. Biologically noteworthy BG-P_400_-TAT targets among intracellular multiprotein complexes are the NF-kB complex, Survivin complex, Bcl-2 complex, and VEGF-A complex (Figure 8A) integrated with signal transduction pathways of EGF, FGF1, AR, estradiol, TNFα, and IL-1β (Figure 8B).

## Discussion

Elevated expression of integrin αvβ3 is associated with tumor aggressiveness and is known to confer treatment resistance in neuroblastoma 31. We have previously shown the synthesis and scaleup of BG-P_400_-TAT and assessed its anti-cancer activity active against in SCID mice xenografted with SK-N-FI cells 13. Here, we investigated the targetability and anti-tumor activity of BG-P_400_-TAT in SCID and NSG mice with subcutaneous neuroblastoma xenografts using a three neuroblastoma cells. In NSG mice, there was a significant increase in tumorigenesis in SK-N-FI, SMS-KCN and SMS-KANR (***P< 0.001) xenografts when compared with SCID mice. The difference might be related to the mice strains. In NSG mice, the immune system is deficient in T cells, B cells, and NK cells, but in SCID mice the immune system is active with B cells and NK cells. So, xenografts are more successful in NSG mice than in SCID mice. The use of xenografts in immunodeficient mice prevents us from assessing important clinical aspects of this therapy such as intra-tumoral changes.

Neuroblastoma is an aggressive cancer among childhood cancers, and approximately 90% of neuroblastomas express NET and integrin αvβ3. BG has been shown to target neuroendocrine tumors 32. Horwacik and Rokita showed that targeting αvβ3 integrin can control cancer biology such as tumor angiogenesis, growth, and metastasis in neuroblastoma 33.

In the present study BG-P_400_-TAT significantly decreased tumor growth rate in SK-N-FI, SMS-KCN and SMS-KANR in SCID mice and NSG mice when compared to their respective controls. Further, the histopathological studies showed high levels of necrosis and hyalinization in treated groups, whereas the controls had viable cells in the xenografts. Our molecular genomic studies of BG-P_400_-TAT have shown that it upregulates tumor apoptosis and pro-apoptosis gene pathways and downregulates tumor survival pathways.

The norepinephrine/catecholamine transporter is essential for norepinephrine uptake at synaptic terminals and adrenal chromaffin cells. In neuroendocrine tumors, the NET is very active and can be exploited for diagnostic imaging purposes and/or therapy with localized radiotherapy. Integrin αvβ3 is generously expressed by and/or activated on cancer cells, but not by nonmalignant, nondividing cells. Integrin αvβ3 is critical to cell-cell interactions, tumor cell motility, extracellular matrix protein-cell interactions and tumor-relevant angiogenesis 34. BG-P_400_-TAT is a dual targeting agent, recognizing the NET system and the thyrointegrin αvβ3 receptor, both of which are overexpressed on the cell surface by neuroblastoma and other neuroendocrine tumors. The addition of BG maximally concentrated the thyrointegrin αvβ3 antagonist in neuroblastoma. A function of the PEG400 linker is to restrict the molecule to the cell surface and prevent nuclear translocation 13.

In addition, the PK and the biodistribution profile of BG-P_400_-TAT supports its plausible application as an anticancer therapeutic for neuroblastoma and other types of cancers. Further the RNAseq genome-wide expression profiling experiments in SKNAS reported BG-P_400_-TAT targets intracellular multiprotein complexes NF-kB, Survivin, Bcl-2 and VEGF-A complex and integrated with signal transduction pathways of EGF, FGF1, AR, estradiol, TNFα, and IL-1β. Thus, the novel composition of the BG-P_400_-TAT molecule improves targeting and theranostic outcome in these preclinical studies and offers new hope for children with neuroblastoma.

## Significance Statement

BG-P_400_-TAT is a novel chemical entity that affects the transcription of a substantial panel of genes relevant to cancer cell proliferation, apoptosis, and cancer-linked angiogenesis mediated via its TAT binding to integrin αvβ3, a plasma membrane protein preferentially expressed by cancer cells while BG binds to the norepinephrine transporter expressed in neuroblastoma and neuroendocrine cancers.

BG-P_400_-TAT targets key gene pathways in neuroblastoma via its downregulation of CDKs, MEK, mTOR, PD-L1 genes and other tumor survival genes while upregulating pro-apoptosis genes.
